# Prevalence of head lice and hygiene practices among women over twelve years of age in Sindh, Balochistan, and North West Frontier Province: National Health Survey of Pakistan, 1990-1994

**DOI:** 10.1186/1756-3305-4-11

**Published:** 2011-02-02

**Authors:** Sadia Mahmud, Gregory Pappas, Wilbur C Hadden

**Affiliations:** 1 Department of Community Health Sciences, Aga Khan University, Stadium Road, P O Box 3500, 74800 Karachi, Pakistan; 2 Global Health Consultant, 910 M Street NW #126, Washington DC 20001 USA; 3 Senior Research Scientist, Center for Innovation, Department of Sociology, University of Maryland, College Park, MD 20742 USA

## Abstract

**Background:**

Head lice infestation is an infection of the scalp and skin which causes blood loss, discomfort, and social and psychological distress with the possibility of secondary bacterial infections occurring at scratch sites. In Pakistan, although some small scale studies have been conducted to investigate prevalence of head lice in school children and the general population, no population based estimates have been reported. The National Health Survey of Pakistan (NHSP 1990 - 94) was a nationally representative health examination survey of the Pakistani population. The NHSP is the first population based household survey to collect data on the prevalence of head lice in adult women in Pakistan. In this paper we use data from the NHSP to present an epidemiological profile of personal hygiene practices and head lice infestation among women aged 12 years or older in three provinces of Pakistan; Balochistan, Sindh and North West Frontier Province (NWFP).

**Results:**

Overall about 7% women aged 12 years and older suffered from head lice infestation. Multivariable logistic regression analysis identified factors independently associated with presence of head lice. Age less than 16 years and crowding at home were associated with higher infestation-rates. The impact of household socio-economic status on infestation rates among women was different in urban and rural settings; urban women with low socio-economic status were more vulnerable than similar women in rural settings. Bathing infrequently in summer was associated with higher prevalence rates only in Sindh, possibly due to the fact that among the three provinces Sindh has a hotter and more humid summer.

**Conclusions:**

The results of our analysis of NHSP indicate high levels of head lice infestation among girls and women in the three Provinces. The epidemiological profile of hygienic practices of women indicated that NWFP and Balochistan as compared to Sindh, and rural as compared to urban areas were less developed with respect to access to water supply and soap for maintaining personal hygiene. Simple and cost-effective measures such as provision of water and soap, and improving awareness regarding maintaining personal hygiene can contribute significantly towards improving public health status of the women in Pakistan.

## Background

Pediculosis capitis, also known as head lice infestation, is caused by *Pediculus humanus capitis *an ectoparasite of man found on the hair and scalp [1]. Most of the reported studies on epidemiology of head lice are restricted to school populations. An epidemiological survey conducted in school children aged 8 -- 16 years in Peshawar, Pakistan from April -- December 1986 identified an overall prevalence of 46%, with girls having a higher prevalence rate (49%) than boys (40%) [2]. The infestation rate decreased as a linear function of age in both sexes, and increased with increasing crowding at home. A survey conducted among 6 to 15 years old urban and rural elementary school children in southern Israel indicated that 55% of the children were infested with head lice, the lowest rate (37%) was observed in children living in an urban high socio-economic neighborhood. Crowding was the main factor contributing to variation in the rates of infestation. An intervention program initiated immediately after the first examination including health education for children and parents, and free medicated shampoo provided for each child detected positive resulted in a significant reduction in head lice infestation one month later [3]. The overall prevalence of head lice infestation in preschool and school children in Korea, estimated from a sample of more than ten thousand children in 9 urban and 8 rural areas, was found to be 24.4% with rural children having a higher (58.9%) infestation rate than urban children (14.4%). The infestation rate increased gradually from the age of 6 years, reached a plateau between 9 - 12 years of age and then slowly decreased with increasing age [4].

In southeast of Iran the estimated prevalence rate among urban primary school girls aged 6--14 years was about 27% [5]. A survey conducted among school children aged 5--19 years, from a low socio-economic area of Tabriz city, Iran in 2006 reported that all the infested cases were seen among girls and there was no infestation among boys; girls aged 10 - 14 years had the highest (6.5%) and those aged 15 - 18 years had the lowest infestation rate (1.6%) [6]. The rate of head lice infestation estimated from a sample of primary school girls (aged 6 - 14 years) in two districts within the Gaza Governorate was 14.1%, with an additional 35% of girls having nits (eggs) only. The prevalence of infestation was high among 6 - 12 years old girls, a sharp decline in infestation was observed after 12 years of age with no lice found on 13 to 14 year olds [7].

The prevalence of head lice infestation among the general population of four urban localities in the North West Frontier Province (NWFP) of Pakistan in 1986 was estimated to be 36.7%, with females showing a higher prevalence (41.5%) than males (27.7%) [8]. Crowding and low level of education were associated with higher rates of infestation. Infestation was high in subjects aged 5 -- 19 years, beyond which it decreased gradually in females and abruptly in males. However, this study was restricted to a few localities in one province (NWFP) of Pakistan and the sampling was not random. Hence the results cannot be generalized. A cross-sectional survey conducted in a representative population of an urban slum and a fishing community in Brazil in 2001 indicated a prevalence of pediculosis capitis as 43.4% in the slum and 28.1% in the fishing community. Children aged 10--14 years and females were most frequently affected; 57.6% women in the urban slum and 41.2% in the fishing community were infested with pediculosis capitis [9].

The National Health Survey of Pakistan (NHSP 1990 - 94) was a nationally representative comprehensive health examination survey of the Pakistani population [10]. In this paper we use the NHSP to present an epidemiological profile of personal hygiene practices and head lice infestation among women aged 12 years or older in three provinces of Pakistan. NHSP is the first population based household survey of women (aged 12 years or older) that provides data to establish prevalence of head lice in Pakistan. Personal hygiene practices and hygienic prevailing conditions are essential with regards to disease prevention and maintaining good health. Personal hygiene practices of adolescent women need special attention as these set the stage for their reproductive health and lifestyle in later life [11]. In this paper we also examine the association of head lice infestation with personal hygiene practices of the Pakistani women.

## Methods

The National Health Survey of Pakistan (NHSP) (1990 -- 1994) was a collaborative effort of the Pakistan Medical Research Council (PMRC), Federal Bureau of Statistics (FBS) and National Center of Health Statistics (NCHS) of the Centers for Disease Control (CDC), US Public Health Service. The objective of NHSP was to obtain accurate health statistics and a health profile of the people of Pakistan by collecting a nationally representative sample. During the planning phase of NHSP an institutional Review Board was constituted, that ensured the survey was built on sound ethical foundations and guidelines. The procedures of informed consent and confidentiality were discussed and approved [11].

A two-stage stratified cluster sampling strategy was adopted to collect the data. The target population was divided into eight strata, comprising of the urban and rural areas (according to 1981 population census) of the four provinces (Punjab, Sindh, NWFP and Balochistan). Enumeration blocks consisting of approximately 200 -- 250 households in the urban strata, and villages in the rural strata were considered as primary sampling units (PSU). From each stratum PSUs were selected with probability proportional to size with respect to the number of households in the urban strata, and village populations as enumerated in 1981 population census in the rural strata. From each PSU a sample of 30 households was selected using systematic sampling by taking a random start and a sampling interval. A total of 80 PSUs were selected, out of these 32 were drawn from urban and 48 from rural areas. All subjects residing in the 2400 households were selected for the study. Overall, 92.4% of selected households participated in the survey and 18, 315 subjects were interviewed. Of these 5655 were women aged 12 years or older. The data were collected in two major parts; household interview survey and health examination survey. A battery of questions concerning the health situation of the household and individual members was administered at home. The responses were provided by a responsible member, usually a mother. Health examinations were conducted on all members of selected households in a mobile examination center. Well-defined questionnaires and protocols were developed and used during the examination [11]. All subjects aged 5 years and older were interviewed concerning personal hygiene, that included the method of cleaning after using the toilet, frequency of bathing in summer (number of times per week) and in winter (number of times per month) respectively, place of bathing and wearing of shoes inside and outside home.

Presence of head lice for NHSP was determined by use of a standardized protocol derived from clinical methods. A new lice comb was provided to each female over the age of twelve who was instructed by a survey technician to draw the comb through the entire lengthen of her hair three times, starting at the scalp at the forehead and moving behind the ear. The comb was then examined under a hand magnifying glass with the aid of a desk lamp and viable nits were counted by the technician. The survey technicians were women trained to the level of Lady Health Workers, that is, two years of basic health education. As part of their training the survey technicians were instructed in administration of the head lice protocol and given basic training in identification viable nits. There was no secondary verification by laboratory or clinical specialists. As such the survey method is similar but not identical to clinical diagnosis of head lice and likely underestimated the prevalence of lice.

The analysis reported in this paper is of three selected Provinces in Pakistan, Sindh, NWFP, and Balochistan. On preliminary analysis it was discovered that Punjab had much higher levels of head lice. Given what is scientifically known about head lice and with investigation of the association found in the data, this effect persisted and was unexplained. For this analysis we have analyzed the three provinces that had similar levels of head lice together. Interviews with those who collected the data in NHSP suggested that methodological differences between the different teams that collected data in each province may explain the provincial difference. The number of women aged 12 years or older in the NHSP data from the three provinces clustered in 40 PSUs was 2788. Data were missing for <6% of women on the variable "number of head lice" and for <7% on socio-demographic characteristics & personal hygiene practices. Women with missing data were excluded from the analysis.

Statistical analysis was conducted with SAS 9.1 software using *proc surveyfreq *and *proc surveylogistic *procedures that adjust for the complex sample design of NHSP [12]. These procedures use the Taylor series expansion method to estimate sampling errors of estimators based on complex sample designs [13]. In Pakistan the population is very unequally distributed among the provinces. In order to ensure that each province was represented in NHSP, the sampling fractions of less populous provinces were larger than those for more populous provinces [14]. Statistical weights were used to adjust for different sampling fractions and make estimates representative of the population of the three provinces.

For NHSP data Hadden etal. [14] developed a measure of household economic status based on possession of durable goods using a principle component analysis. In each household a responsible person was asked about the ownership of household goods including an iron, fan, radio, tape recorder, television, refrigerator, VCR, air conditioner, motorcycle, and a car or a tractor. In the principle component analysis that they conducted, Hadden etal. [14] showed that scores based on the first component explain 36% of the total variance and are highly correlated (*r = 0.98*) with a simple count of the number of durable goods owned by each household. They used this count to divide the population into three levels of household economic status; *low, middle *and *high *corresponding to ownership of 0 - 2, 3 - 5 and 6 or more durable goods respectively. Using the count of the number of durable goods as a continuous variable we conducted quartile analysis in logistic regression to examine the scale of this variable in the model (with the presence of head lice as a binary outcome variable) [15]. Our analysis suggested three categories (0 - 1, 2 - 3 & ≥4) for the number of durable goods as a measure of household economic status. In this paper we refer to the economic status of households owning 0 - 1, 2 - 3 and ≥4 number durable goods as *low, middle *and *high *respectively.

In addition we examined the scale of the variables, 'number of baths a woman takes per week in summer' and 'per month in winter' using quartile analysis in logistic regression that indicated a binary scale for both frequency of summer bathing (<3, ≥3 per week) and that of winter bathing (<5, ≥5 per month). Similarly scale examination of the variable 'average number of persons per room' in a household indicated a binary scale (<3.5, ≥3.5).

## Results

To estimate the prevalence of head lice infestation in women aged 12 years or older we categorized head lice as "none", "mild" and "severe" infestation corresponding to 0, 1 - 3, >3 number of nits observed respectively on the special combs women passed through their hair. Overall the percentage (standard error) of mild and severe infestation was 6.4 (1.2) and 0.8(0.2) respectively (n = 2606). In the urban areas 4.9% (1.5%) women had mild and 0.5% (0.3%) had severe infestation, whereas in the rural areas 7.4% (1.8%) women had mild and 1.0% (0.3%) had severe infestation respectively.

Descriptive analysis of socio-demographic characteristics of women indicated that 38% women were in the age range from 16 to 29 years, whereas about 14% were younger than 16 years. Majority of the women were married (62%) and an alarming majority (78%) were illiterate. About 27% of the women belonged to low and 44% belonged to high economic status households. About 50% of the women lived in crowded households having more than 3.5 persons per room.

Table 1 reports the prevalence of head lice by urban or rural status disaggregated with respect to major socio-demographic characteristics. Crude prevalence estimates were similar for the urban strata in the three provinces, however in the rural strata more women had lice infestation in Sindh (10.7%) as compared to 7.6% in NWFP and only 3.7% in Balochistan. With increasing age head lice infestation decreased both in the urban and the rural women. In the rural areas never married women had a higher prevalence of head lice relative to ever married women, but the association between marital status and lice infestation was not significant in the urban area. Head lice infestation was more prevalent among women from households belonging to low economic status both in the urban and in the rural areas.

**Table 1:** Prevalence of head lice^1 ^among Pakistan urban and rural women, aged 12 years and older, by socio-demographic characteristics; National Health Survey of Pakistan (1990 - 94) Sindh, Balochistan, and NWFP Provinces.

	Urban		Rural	
	**Mild**	**Severe**	**Mild**	**Severe**
	
	**Percent (SE)**			
**Variables**				

	**(n = 1015)**		**(n = 1591)**	
**Province**				
Sindh	5.0(1.8)	0.2(0.2)	10.1(4.2)	0.6(0.4)
NWFP	4.8(1.6)	2.1(2.0)	6.6(1.2)	1.0(0.6)
Balochistan	4.5(3.6)	1.3(1.3)	1.8(0.5)	1.9(0.7)
				
**Age categories**				
12 -- 15 yrs	9.4(4.0)	0.9(0.9) **	14.5(3.4)	2.8(1.4) **
16 - 29 yrs	6.0(1.7)	0.3(0.2)	5.4(1.2)	0.8(0.4)
30 -- 44 yrs	3.0(1.3)	0.7(0.5)	6.6(2.2)	1.0(0.5)
≥45 yrs	2.4(1.3)	0.2(0.2)	7.3(2.6)	0.3(0.2)
	**(n = 979)**		**(n = 1476)**	
**Marital Status**				
Never married	6.2(2.4)	0.4(0.4)	12.7(2.9)	2.1(0.9) *
Married	4.2(1.8)	0.6(0.4)	6.2(1.5)	0.7(0.3)
Widow/sep/div	4.3(3.2)	0.2(0.2)	7.5(4.8)	0.7(0.5)
	**(n = 967)**		**(n = 1461)**	
**Education**				
Illiterate	4.9(1.9)	0.7(0.5) ±	7.6(2.0)	1.1(0.4) ±
Less than matric	5.9(2.4)	0.2(0.2)	11.4(4.2)	-
Matric and above	3.9(1.1)	-	6.3(5.9)	-
	**(n = 975)**		**(n = 1518)**	
**Ownership of** **Durable goods**				
None to 1	17.9(7.7)	1.5(1.5)*	9.5(2.8)	1.2(0.4)*
2 - 3	4.7(2.4)	0.7(0.5)	8.4(2.0)	0.7(0.6)
4 or more	3.8(1.5)	0.1(0.1)	4.0(0.8)	1.1(0.6)
	**(n = 987)**		**(n = 1572)**	
**Crowding** **(# of persons per room)**				
<3.5	3.3(0.9)	0.5(0.3)	6.3(1.7)	0.7(0.3)
≥3.5	6.6(2.6)	0.5(0.5)	8.4(2.0)	1.3(0.4)

1. Head lice is categorized as "None, Mild and Severe infestation"

* 0.01 < p-value < 0.05, ** p-value < 0.01 (Rao-Scott likelihood ratio test, association of head lice with the variable within rural and urban).

± No test was computed as one cell had zero frequency.

Tables 2 reports personal hygiene practices of women, and their use of prescription medication to control head or body lice (during the past 12 months), by province and urban/rural status. About 7% women in Sindh, 4% in NWFP and 13% in Balochistan had used prescription medication to control body or head lice during the past 12 months. Twenty percent women in NWFP, 10.5% in Balochistan and only about 2% in Sindh had ever used dichloro-diphenyl-trichloro-ethane (DDT) for head lice. About one half of the urban and one third of the rural women always wore shoes at home. Ninety four percent urban women and about 79% rural women always wore shoes outside home.

**Table 2:** Personal hygiene and medication (for lice) use in Pakistan women, aged 12 years and older, by province and urban/rural status; National Health Survey of Pakistan, 1990 - 94 Sindh, Balochistan, and NWFP Provinces.

	Sindh	NWFP	Balochistan	Urban	Rural
		
	Percent (SE)				
Variables					
**Used medication^1 ^**(n = 2598)					
No (n = 2410)	93.4(2.2)	95.9(1.3)	86.8 (7.9)	93.8(2.2)	93.3(2.1)
Yes (n = 188)	6.6(2.2)	4.1(1.3)	13.2 (7.9)	6.2(2.2)	6.7(2.1)
					
**Ever Used DDT **(n = 2596) **for head lice**					
No (n = 2342)	97.8 (0.6)	80.0 (2.5)	89.5(5.6)	97.5(0.5)	87.3(1.7)
Yes (n = 254)	2.2 (0.6)	20.0 (2.5)	10.5 (5.6)	2.5(0.5)	12.7(1.7)
					
**Cleaning after using toilet **(n = 2601)					
Water (n = 2156)	97.6 (1.3)	48.2 (9.3)	98.0 (0.9)	99.3 (0.3)	70.9(5.1)
Stone/clay (n = 406)	2.1 (1.3)	47.7 (8.8)	1.7(1.0)	0.4 (0.2)	26.9(4.9)
Paper/other (n = 39)	0.3(0.1)	4.0 (2.1)	0.3 (0.2)	0.2 (0.1)	2.2(1.1)
					
**Clean hands after using toilet **(n = 2601)					
Soap & water (n = 1265)	64.0 (5.7)	29.0 (5.9)	40.3 (12.7)	87.6(3.7)	25.8(4.9)
Water (n = 1102)	20.3 (5.3)	70.9 (5.8)	59.0 (12.7)	10.5(3.3)	59.8(5.6)
Do not usually/Stone/clay/ grass/sand/other (n = 234)	15.7 (3.7)	0.2 (0.1)	0.7 (0.4)	2.0(1.6)	14.4(3.6)
					
**Number of baths** **in summer (per week) **(n = 2600)					
≥3 (n = 2324)	93.8 (3.5)	88.9 (1.6)	80.4 (12.1)	97.3(0.6)	86.5(4.2)
<3 (n = 276)	6.2 (3.5)	11.1 (1.6)	19.6 (12.1)	2.7(0.6)	13.5(4.2)
					
**Number of baths** **in winter (per month) **(n = 2599)					
≥5 (n = 1153)	67.2(5.0)	24.3(4.3)	27.9(14.6)	71.6(5.0)	35.2(5.8)
<5 (n = 1446)	32.8(5.0)	75.7(4.3)	72.1(14.6)	28.4(5.0)	64.8(5.8)
					
**Where do you bathe? **(n = 2600)					
Home (n = 2589)	99.8 (0.2)	99.0 (0.7)	100.0 (0)	99.95(0.04)	99.3(0.4)
Other ^2 ^(n = 11)	0.2 (0.2)	1.0 (0.7)	-	0.04(0.04)	0.7(0.4)
					
**Wear shoes at home **(n = 2600)					
Always (n = 1029)	31.6 (5.0)	65.1 (6.8)	15.0 (3.2)	47.8(6.2)	35.1(4.4)
Sometimes (n = 1473)	62.9 (5.0)	33.3 (6.7)	81.0 (3.8)	49.4(5.9)	59.7(4.7)
Never (n = 98)	5.5 (2.3)	1.6 (0.5)	4.0 (2.6)	2.8(1.1)	5.1(2.2)
					
**Wear shoes outside home **(n = 2600)					
Always (n = 2239)	79.3 (3.4)	96.8 (1.0)	85.3 (8.4)	94.0(2.3)	79.4(3.5)
Sometimes (n = 349)	20.1 (3.3)	2.5 (1.0)	14.4 (8.3)	5.9(2.3)	19.7(3.4)
Never (n = 12)	0.6 (0.3)	0.7 (0.3)	0.2 (0.2)	0.1(0.1)	0.9(0.3)

1. Prescription medication to control body or head lice, during the past 12 months

2. Mosque/Barber’s hammam/Canal/Pond/Other

Majority of the women used water to clean themselves after using toilet that is a hallmark of a Muslim culture. In urban areas 88% and in rural areas only 26% women used soap & water for cleaning hands after using toilet. In NWFP & Balochistan less than half of the women used soap & water for cleaning hands after using toilet. Majority of the women in the urban areas bathed frequently in summer (97%) and in winter (72%). However, in the rural areas about 86% women bathed frequently in summer and only 35% in winter respectively. In NWFP and Balochistan only about one-fourth of the women bathed frequently in winter (Table 2).

Table 3 presents the prevalence of head lice disaggregated with respect to personal hygiene practices, and use of prescription medication and DDT to control lice. There was no significant difference in the prevalence of head lice between women who had used prescription medication to control head or body lice (during the past 12 months) and those who did not. Similarly ever use of DDT for head lice did not have any impact on prevalence of head lice. Women who bathed less than 3 times per week in summer had a significantly higher prevalence of head lice (16.2% mild and 1.8% severe) relative to those who bathed more frequently (5.4% mild and 0.7% severe). Similarly, women who bathed less than 5 times per month in winter had a higher prevalence of head lice (7.8% mild and 1.2% severe) relative to women who bathed more frequently (5.0% mild and 0.3% severe). About 24% women who never wore shoes at home had mild head lice infestation compared to only about 5% among women who always wore shoes at home.

**Table 3:** Prevalence of head lice^1 ^among Pakistan women, aged 12 years and older, by personal hygiene practices and use of medication/DDT to control lice; National Health Survey of Pakistan, 1990 - 94 Sindh, Balochistan, and NWFP Provinces.

	Mild	Severe
	
	Percent (SE)	
Variables		
**Used medication^2 ^**(n = 2598)		
No	6.4(1.3)	0.7(0.2)
Yes	5.9(2.2)	1.5(1.0)
		
**Ever Used DDT **(n = 2596) **for head lice**		
No	6.4(1.3)	0.7(0.2)
Yes	6.0(1.4)	1.8(0.8)
		
**Cleaning after using toilet **(n = 2601)		
Water	6.5(1.4)	0.7(0.2) ±
Stone/clay	6.0(1.9)	1.1(0.6)
Paper/other	7.9(2.8)	-
		
**Clean hands after using toilet **(n = 2601)		
Soap & water	5.1(1.3)	0.4(0.2)
Water	7.2(2.0)	1.2(0.4)
Do not usually/Stone/ clay/grass/sand/other	10.2(4.6)	1.3(1.0)
		
**Number of baths** **in summer (per week) **(n = 2600)		
≥3	5.4(1.1)	0.7(0.2) **
<3	16.2(6.1)	1.8(0.9)
		
**Number of baths** **in winter (per month) **(n = 2599)		
≥5	5.0(1.2)	0.3(0.2) *
<5	7.8(1.7)	1.2(0.4)
		
**Where do you bathe? **(n = 2600)		
Home	6.4(1.2)	0.7(0.2)
Other ^3^	-	7.6(6.5)
		
**Wear shoes at home **(n = 2600)		
Always	4.8(0.9)	0.3(0.2) ±
Sometimes	6.2(1.2)	1.2(0.3)
Never	23.6(8.8)	-
		
**Wear shoes outside home **(n = 2600)		
Always	5.6(0.9)	0.7(0.3) ±
Sometimes	10.9(4.6)	1.4(0.6)
Never	16.2(9.4)	-

1. Head lice is categorized as "None, Mild and Severe infestation"

2. Prescription medication to control body or head lice, during the past 12 months

3. Mosque/Barber's hammam/Canal/Pond/Other

* 0.01 < p-value < 0.05, ** p-value < 0.01 (Rao-Scott likelihood ratio test).

± No test was computed as one cell had zero frequency.

For conducting logistic regression analysis, head lice infestation was dichotomized as 'absent' versus 'present'. Crude odds ratios for association of head lice with socio-demographics, personal hygiene practices and use of medication/DDT to control lice are presented in Table 4 &5 respectively. Table 6 presents the results of multivariable logistic regression analysis with adjusted odds ratios that provide measures of independent association of head lice in women with their socio-demographics and personal hygiene practices. Age less than 16 years (OR_adj _= 2.4, 95% CI: 1.6, 3.6) and crowding in the household (OR_adj _= 1.5, 95% CI: 1.1, 2.1) were independently associated with head lice infestation in women. Considering the practice of wearing shoes as a proxy variable for personal hygiene, the multivariable analysis indicated a marginal association of wearing shoes at home with head lice infestation (OR_adj _= 1.5, 95% CI: 0.9, 2.4). There was a significant interaction between women's economic and urban/rural status. Urban women with low economic status were 7.6 times (OR_adj_= 7.6, 95% CI: 2.7, 21.2) more likely, and rural women with low economic status were only about twice as likely to have head lice (OR_adj _= 2.1, 95% CI: 0.6, 7.1) relative to urban women with high economic status. In addition, there was a significant interaction between infrequent bathing in summer and the province where a woman resided. In Balochistan bathing <3 times per week in summer did not have a significant effect on head lice infestation (OR_adj _= 0.9, 95% CI: 0.3, 3.2). However, Sindhi women who bathed <3 times per week were about 9 times more at risk than Balochi women who bathed frequently (OR_adj _= 9.3, 95% CI: 3.8, 23.0). In NWFP both frequent bathers (OR_adj _= 2.0, 95% CI: 0.9, 4.8) and infrequent bathers (OR_adj _= 1.7, 95% CI: 0.5, 6.0) were about twice as likely to suffer from head lice infestation as women who bathed frequently in Balochistan.

**Table 4:** Univariate logistic regression and crude odds ratio for association of head lice^1 ^in women aged 12 years and older with socio-demographics and urban/rural status; National Health Survey of Pakistan, 1990 - 94 Sindh, Balochistan, and NWFP (n = 2321).

Variables	Crude Odds Ratio	95% CI
**Age categories**		
12 - 15 yrs	2.7	(1.5, 4.8)
16 - 29 yrs	1.0	(0.6, 1.8)
30 - 44 yrs	0.9	(0.5, 1.5)
≥45 yrs	1.0	-
		
**Marital Status**		
Married	1.0	-
Never married/ Wid/Div/Sep	1.7	(1.2, 2.4)
		
**Education**		
Illiterate	1.2	(0.7, 2.2)
Less than Matric/ Matric & above	1.0	-
		
**Area**		
Urban	1.0	-
Rural	1.8	(0.8, 3.9)
		
**Province**		
Sindh	1.8	(0.8, 4.4)
NWFP	1.8	(0.9, 3.8)
Balochistan	1.0	-
		
**Ownership of Durable goods**		
None to 1	2.9	(1.4, 5.9)
2 - 3	1.9	(1.0, 3.5)
4 or more	1.0	-
		
**Crowding** **(# of persons per room)**		
<3.5	1.0	-
≥3.5	1.7	(1.1, 2.5)

1.Present versus absent

**Table 5:** Univariate logistic regression and crude odds ratio for association of head lice^1 ^in women aged 12 years and older with personal hygiene and use of medication/DDT to control lice; National Health Survey of Pakistan, 1990 - 94 Sindh, Balochistan, and NWFP Provinces (n = 2321).

Variables	Crude Odds Ratio	95% CI
**Used medication^2^**		
No	1.0	-
Yes	1.1	(0.5, 2.1)
		
**Ever Used DDT** **for head lice**		
No	1.0	-
Yes	1.2	(0.6, 2.1)
		
**Number of baths** **in summer (per week)**		
≥3	1.0	-
<3	3.1	(1.3, 7.4)
		
**Number of baths** **in winter (per month)**		
≥5	1.0	-
<5	1.9	(1.1, 3.2)
		
**Wear shoes at home**		
Always	1.0	-
Sometimes/Never	1.8	(1.2, 2.9)
		
**Wear shoes outside home**		
Always	1.0	-
Sometimes/Never	2.3	(1.1, 5.0)

1. Present versus absent

2. Prescription medication to control body or head lice, during the past 12 months.

3. Odds ratio is computed for an increase of every one bath/week.

4. Odds ratio is computed for an increase of every one bath/month.

5. Mosque/Barber's hamman/Canal/Pond/Other

**Table 6:** Multivariable logistic regression model and adjusted odds ratio for association of head lice^1 ^in women aged 12 years and older with socio-demographics and personal hygiene; National Health Survey of Pakistan, 1990 - 94 Sindh, Balochistan, and NWFP Provinces (n = 2321).

Variables			
	Adjusted	95% CI	
	Odds Ratio	Lower limit	Upper limit
**Age categories**			
12 - 15 yrs	2.4	1.6	3.6
≥16 yrs	1.0	-	
			
**Crowding (# of persons per room)**			
<3.5	1.0	-	
≥3.5	1.5	1.1	2.1
			
**Wear shoes at home**			
Always	1.0	-	
Sometimes/Never	1.5	0.9	2.4
			
Urban/rural area * ownership of durable goods			
**Living in urban area & ownership of durable goods**			
4 or more	1.0	-	
2 - 3	1.4	0.4	5.1
0 - 1	7.6	2.7	21.2
			
**Living in rural area & ownership of durable goods**			
4 or more	1.4	0.5	4.4
2 - 3	2.0	0.6	6.6
0 - 1	2.1	0.6	7.1
			
Province * Infrequent bathing in summer (number of baths per week			
**Balochistan & number of baths per weeks**			
≥3	1.0	-	
<3	0.9	0.3	3.2
			
**Sindh & number of baths per weeks**			
≥3	1.5	0.5	4.6
<3	9.3	3.8	23.0
			
**NWFP & number of baths per weeks**			
≥3	2.0	0.9	4.8
<3	1.7	0.5	6.0
			
**Marital Status **§			
Married	1.0	-	
Never married/ Wid/Div/Sep	1.3	0.8	2.2
			
**Number of baths **§ **in winter (per month)**			
≥5	1.0	-	
<5	1.3	0.8	1.9

§ These variables are included in the model because they confound the effect of other variables

## Discussion

In this paper a nationally representative sample of women aged twelve years and older, from NHSP (1990 -- 1994), reveals high levels of lice infestation among girls and women in the three Provinces that were studied. The results of this study indicate that NWFP and Balochistan lag behind Sindh in access to water supply and basic commodities such as soap for maintaining personal hygiene. Lack of awareness regarding appropriate hygienic practices in the former two provinces may also be a reason for their women's inadequate personal hygiene status. In addition rural areas appear to be much less developed as compared to urban areas with respect to above mentioned needs of the population. The study presented here did not use data from Punjab because of problems with data from that province in the lice portion of NHSP.

Although it is not known to be a vector of any disease, head lice infestation causes blood loss, irritation, discomfort, and social and psychological distress. Secondary bacterial infections can occur at scratch sites [1,2]. However, with regards to blood loss that can result from head lice Spear etal [16] showed that for an average infestation of 30 lice per head the blood loss is too minor to be of any clinically significance. Prevalence estimates from our analysis of NHSP are much smaller than the 42% infestation rate among females reported from a survey conducted among the general population in some urban localities in NWFP [8]. This study was conducted in selected localities and the sample was not representative of NWFP or Pakistan. However, the difference in the prevalence estimates between this survey and the NHSP could also be due to different protocols for detecting head lice in the two surveys. In the survey conducted in reference [8] screening was done by visual inspection and those having or suspected of having lice were subjected to combing of the head with a fine-toothed comb for about two minutes onto a white paper sheet and number of head lice counted. Using the same method for detecting head lice Suleman and Fatima [2] reported an infestation rate of 49% among 8 - 16 year old school girls in Peshawar, the capital of NWFP. Infestation rates in a comparable age group of girls (12 -- 15 years) computed from NHSP in our study were considerably smaller; 10% in the urban and 17% in the rural areas.

Our prevalence estimates are also smaller than those reported for school girls in a survey conducted in southeast Iran as 27% among 6 -- 14 year olds [5]. In the latter survey hair inspection was carried out by dividing the scalp into four sections with a rat-tail comb and hair clips in a well lighted area for about 3 - 5 minutes, those suspected of having lice were combed with a fine-toothed comb for about 7 minutes over a white paper and number of head lice counted. Hence, it seems that among other factors contributing to variation in head lice infestation rates across different surveys, different methods for detecting presence of head lice can also lead to different prevalence estimates. In a survey conducted in a low socio-economic area of Tabriz city, Iran, head lice was diagnosed by clinical inspection of scalp and hair and by using a manual magnifier, and reported a lower infestation rate of 6.5% among girls aged 10 - 14 years and 1.6% among those aged 15 - 18 years [6]. While our study uses a rather simple methodology for diagnosing head lice, there is not a globally accepted epidemiological definition. The highly standardized protocol used in NHSP (three passes of a comb through the hair) would be expected to be less sensitive than the two minute combing method.

Our multivariable logistic regression analysis of NHSP identifies factors independently associated with presence of head lice. Women younger than 16 years are twice as likely to suffer from lice infestation as older women. Other studies have identified a similar trend of decreasing infestation rates among women with increasing age after 12 years [2,4,6,8]. Usually older women are more conscious and aware of personal hygiene and self care. Also, young adolescent women are more prone to behaviors such as close contact and touching with friends and playmates. Moreover, we found that crowding in a household is independently associated with head lice infestation in women. This is as expected since crowding facilitates spread of lice from one person to another. Various other studies have reported that higher head lice infestation rates are associated with increasing crowding at home [2,3,8]. Our study results also indicate that the effect of the socio-economic status of a household on infestation rates is different for urban and rural women. Urban women with low SES are at a much higher risk for lice infestation than rural women with low SES. This implies that poverty has a larger effect on head lice infestation in urban settings. A cross-sectional survey conducted in a representative population of an urban slum in Brazil indicated a very high prevalence of pediculosis capitis (58%) among women [9]. Furthermore, our analysis indicated that bathing infrequently in summer was associated with higher prevalence rates only in Sindh. Among the three provinces Sindh has a hot and humid summer; such a climate tends to facilitate head lice infestation. Hence it appears that infrequent bathing in summer puts the women in Sindh at a high risk for head lice infestation due to the climate conditions. We have discussed the limitation of the protocol for detecting head lice in NHSP with regards to its sensitivity that may lead to underestimation of infestation rate. However, the risk factors found in this study are in line with other studies which speak in favor of the validity of the data.

Finally we note that DDT is used for head lice in the country. DDT does not kill louse eggs efficiently and lice are known to develop resistance [17]. Moreover DDT can be toxic to humans; a study found that in utero exposure to background concentrations of DDT is inversely associated with cognitive functioning among preschool children [18]. In her landmark book *Silent Spring *Rachel Carson described how DDT entered the food chain, accumulated in the fatty tissues of animals and human beings and caused cancer and genetic damage [19]. DDT was banned in the USA in the 1970s but is still used in some countries. Use of DDT to control head lice among women in Pakistan needs to be discouraged, and strategies such as maintaining personal hygiene and using fine-toothed combs after washing of hair should be encouraged.

Availability of water is the most obvious enabling condition for maintaining personal hygiene [20]. From the public health policy viewpoint provision of water supply for the population in less developed provinces of NWFP and Balochistan, and in rural areas of all three provinces should be a priority. Washing of hands with soap & water, particularly after defecation and contact with children's faeces when hands get dangerously soiled, is the most effective behavior for prevention of diarrhea as well as that of roundworm and whipworm [20]. Simple and cost-effective measures such as provision of soap, and awareness raising through print and electronic media regarding importance of hand washing can contribute significantly towards improving public health status. A study conducted among school children in Israel showed a significant reduction in head lice infestation one month after health education for children and parents, and providing free medicated shampoo to children detected with head lice [3]. Similar strategies can be adopted for Pakistani women attending schools and health centers and through Lady Health Workers (who visit women in their houses) with special emphasis on adolescent women, and those living in crowded and low socio-economic households.

## Conclusions

The epidemiological profile of hygienic practices of women in the three Provinces studied indicated that NWFP and Balochistan lag behind Sindh in access to water supply and soap for maintaining personal hygiene. Similarly rural areas appeared to be much less developed as compared to urban areas. The results of our analysis of NHSP indicate high levels of head lice infestation among girls and women in the three Provinces. Younger age, household crowding and socio-economic status, and infrequent bathing in summer were independently associated with head lice infestation. DDT is used for head lice in the country; its use needs to be discouraged due to its harmful effects on humans and resistance against eradication of lice. Simple and cost-effective measures such as provision of water and soap, and improving awareness regarding maintaining personal hygiene can contribute significantly towards improving public health status of the women in Pakistan.

## Competing interests

The authors declare that they have no competing interests.

## Authors' contributions

SM participated in the conceptualization of the study, conducted the analysis and drafted the manuscript for the paper.

GP participated in the conceptualization of the study and the acquisition of the data, reviewed the analysis, and participated in the revision of the manuscript.

WCH contributed to the conceptualization and analysis and participated in the revision of the manuscript.

All authors read and approved the final manuscript.
